# Proteomic Analysis of Chikungunya Virus Infected Microgial Cells

**DOI:** 10.1371/journal.pone.0034800

**Published:** 2012-04-13

**Authors:** Bizunesh Abere, Nitwara Wikan, Sukathida Ubol, Prasert Auewarakul, Atchara Paemanee, Suthathip Kittisenachai, Sittiruk Roytrakul, Duncan R. Smith

**Affiliations:** 1 Molecular Pathology Laboratory, Institute of Molecular Biosciences, Mahidol University, Bangkok, Thailand; 2 Department of Microbiology Faculty of Science, Mahidol University, Bangkok, Thailand; 3 Center for Emerging and Neglected Infectious Diseases, Mahidol University, Bangkok, Thailand; 4 Department of Microbiology, Faculty of Medicine, Siriraj Hospital, Mahidol University, Bangkok, Thailand; 5 Genome Institute, National Center for Genetic Engineering and Biotechnology, National Science and Technology Development Agency, Pathumthani, Thailand; Agency for Science, Technology and Research - Singapore Immunology Network, Singapore

## Abstract

Chikungunya virus (CHIKV) is a recently re-emerged public health problem in many countries bordering the Indian Ocean and elsewhere. Chikungunya fever is a relatively self limiting febrile disease, but the consequences of chikungunya fever can include a long lasting, debilitating arthralgia, and occasional neurological involvement has been reported. Macrophages have been implicated as an important cell target of CHIKV with regards to both their role as an immune mediator, as well evidence pointing to long term viral persistence in these cells. Microglial cells are the resident brain macrophages, and so this study sought to define the proteomic changes in a human microglial cell line (CHME-5) in response to CHIKV infection. GeLC-MS/MS analysis of CHIKV infected and mock infected cells identified some 1455 individual proteins, of which 90 proteins, belonging to diverse cellular pathways, were significantly down regulated at a significance level of *p*<0.01. Analysis of the protein profile in response to infection did not support a global inhibition of either normal or IRES-mediated translation, but was consistent with the targeting of specific cellular pathways including those regulating innate antiviral mechanisms.

## Introduction

Chikungunya virus (CHIKV) is the causative agent of the mosquito transmitted disease chikungunya fever and infection of humans with CHIKV results an illness traditionally characterized by high fever, rash, arthritis and an erratic relapsing and incapacitating arthralgia [1], [2]. The disease was first formally described after an outbreak in 1952 in Tanzania [3] and the virus was first isolated from the same outbreak [4].

CHIKV is an enveloped icosahedral, positive single-stranded-RNA virus, belonging to the genus *Alphavirus* in the family *Togaviridae*, and the approximately 11.8 kb genetic material which contains a 5′-methylguanylate cap and a 3′-polyadenylate tail [5] codes for 4 non-structural proteins (nsP1 to nsP4), three structural proteins (capsid, E1 and E2) and two small peptides (E3 and 6K) in two open reading frames [5], [6]. After entry to a host cell by endocytosis [7] and uncoating, the genomic RNA is translated directly into the four non-structural proteins which are encoded by the 5′-two thirds of the genome. These proteins collectively form the replicative enzyme complex which mediates the replication of the viral genome and transcription of a 26S subgenomic RNA which encodes for the structural proteins [6]. The four nonstructural proteins possess methyltransferase and guanyltransferase activity (nsP1), protease, helicase, NTPase and 5′ triphosphatase activity (nsP2), RNA dependent polymerase and adenyltransferase activity (nsP4) while nsP3 is predominantly responsible for synthesis of the minus strand RNA replicative intermediate [6].

There are three lineages of CHIKV the so called West African, East Central and South African (ECSA) and Asian lineages, and as the names imply the first two are predominantly associated with transmission in Africa, while the Asian lineage, which is believed to have diverged from the ECSA lineage between 50 and 300 years ago circulates in Asia [8], [9]. CHIKV is transmitted by the bite of infected mosquitoes of the *Aedes* genus, and in Asia the virus is maintained in an urban transmission cycle between humans and the anthropophilic *Aedes aegypti*, while in African the virus is believed to be maintained in a primarily sylvatic cycle between non-human primates and forest dwelling *Aedes* mosquito species [1], [10].

While CHIKV has been present at low levels in Africa, India and Southeast Asia over the last 50 years [11], it re-emerged into prominence as a major public health concern after the massive outbreak in La Reunion in 2007 and the subsequent outbreaks in India, Sri Lanka, Singapore, Malaysia, and Thailand over the following years [9], [12], [13], [14]. Of particular concern was the adaptation of the virus to *Aedes albopictus* hosts [15] which drove much of the outbreak, as well as led to autochthonous outbreaks in Europe [16], [17] and the possibility of significantly greater spread around the world to countries where *Aedes albopictus* is well established [18].

Chikungunya is classified as an “Old world" *alphavirus* together with Sindbis virus and Semliki Forest virus and these viruses are predominantly arthralgic in presentation, as opposed to the “New world" alphaviruses (such as Venezuelan equine encephalitis virus and Eastern equine encephalitis virus) that are predominantly encephalitic in presentation [6]. However, over the last few decades, there have been increasing number of reports of severe chikungunya with outbreaks in India in the early 1960 s being associated with neurological and hematological complications [19], [20] while recent outbreaks from 2005 onward have been associated with severe encephalitis and meningioencephalitis, as well as an increasing number of fatalities, particularly in the elderly or those with other existing conditions [12], [13], [21], [22], [23].

The tissue tropism of CHIKV in humans is not completely defined, but epithelial, endothelial and fibroblast cells [2] as well as monocytes [24] and in particular monocyte derived macrophages [25] have all been proposed to be involved in the disease pathology, while in the brain both neurons and glial cells have been implicated [22], a finding consistent with the reported neurological involvement [12], [22].

Microglial cells are specialized brain resident macrophages of the central nervous system that are hematopoietic in origin and act as the primary cellular response to invading pathogens or injury [26]. Microglial cells are resident in the parenchyma, and animal model studies suggest that where the CNS is involved in CHIKV infection, it occurs primarily at the level of the meningeal and ependymal layers rather than occurring at the parenchyma [27], and supporting this, where studies have examined microglial cells directly no antigen positive cells were detected [25]. However, activation of microglial cells has been reported in CHIKV infection [28] and while human studies have shown that neonatal encephalopathy lesions were associated primarily with the white matter [27], microglial cells are present in the white matter of normal human brain, although less frequently than in the grey matter [29].

While mouse studies have suggested that microglial cells are refractory to infection [21], our recent study has shown that the transformed microglial cell line CHME-5 is productively highly permissive to CHIKV infection, with the resultant induction of apoptosis [30]. The induction of apoptosis is a common result of infection of mammalian cells with CHIKV [2], [31]. Prior to this however, it is well established that both old and new world alphaviruses induce transcriptional and translational shut off, possibly as a way to avoid antiviral responses during replication [32], [33]. Interestingly, new world alphaviruses use the nsP2 protein to down regulate RNA polymerase I and II transcription while the Old world alphaviruses achieve the same result through the expression of the capsid protein [32]. CHIKV has been shown to induce translational shut off, although the mechanism remains to be explained [34].

As noted earlier, microglial cells are a specialized lineage macrophage [26] and macrophages have been implicated as a long lasting reservoir for CHIKV in animal model studies [25]. In parallel with this we have recently proposed [35] that microglial cells are a long lasting reservoir for the Japanese encephalitis virus (JEV). While the involvement of microglial cells is more pronounced in JEV infections, our study showing that transformed microglial cells are productively infected [30], coupled with the fact that, while rare, neurological involvement in CHIKV infection particularly in neonates has severe consequences [12], [19], [20], [22] prompted us to more fully examine the cellular consequences of CHIKV infection of microglial cells, using transformed cells as a model system.

## Materials and Methods

### Ethics statement

CHIKV E1: 226 V was isolated as part of a study approved by the Mahidol University Institutional Review Board (COA. NO. MU-IRB 2010/251.3018) and by the Ethics Review Board of Pang Nga Hospital, Thailand. Written informed consent was obtained.

### Cells and virus

The human embryonic fetal microglial cell line CHME-5 [36] was kindly provided by Professor Pierre Talbot, Laboratory of Neuroimmunovirology, INRS-Institute, Armand-Frappier, Canada and both CHME-5 and Vero (African green monkey kidney; ATCC Cat No. CCL-81) cells were grown and maintained in Dulbecco's modified eagle's medium (DMEM; Gibco, Invitrogen, Carlsbad, CA) supplemented with 10% or 5% heat-inactivated fetal bovine serum (FBS; Gibco, Invitrogen) respectively and 100 units/ml of penicillin/streptomycin (PAA Laboratories GmbH, Pasching, Austria) at 37°C with 5% CO_2_. CHIKV (ECSA genotype, Thai isolate E1: 226 V) was propagated in Vero cells and infectious virus titer was determined by standard plaque assay on Vero cells.

### Virus infection

A day prior to infection, CHME-5 cells were seeded into cell culture dishes at a density that allowed confluence to be reached within 24 hours. At the time of infection the medium was aspirated immediately before inoculating the cells with CHIKV at the required multiplicity of infection (MOI) diluted in ice-cold serum free medium. Cells were then incubated at 37°C with 5% CO_2_ for 2 hours, with gentle rocking every 20 minutes for 2 hours after which the medium was removed and pre-warmed DMEM supplemented with 10% FBS was added to each dish. The cells were then incubated at 37°C with 5% CO_2_ until required.

### Flow cytometry

For analysis of apoptosis, mock and CHIKV infected cells were collected at day 2 p.i. and washed with ice-cold PBS and were resuspended in binding buffer (BD, Franklin Lakes, NJ), followed by double staining with the addition of 50 µg/ml FITC-conjugated Annexin V and 20 µg/ml propidium iodide. After 15 min, the cells were analyzed by flow cytometry on a FACSCalibur cytometer (BD Biosciences, San Jose, CA) using CELLQuestTM software.

To quantify the infected cells, mock and CHIKV infected cells were harvested and blocked with 10% normal goat serum for 30 min on ice. Cells were washed with 1% BSA followed by fixation in 4% paraformaldehyde at room temperature for 20 min and subsequently permeabilized with 0.2% Triton X-100 in 1% BSA for 10 min at room temperature. Cells were then incubated with a mouse anti-alphavirus monoclonal antibody (Santa Cruz Biotechnology Inc., Santa Cruz, CA) at a dilution of 1∶200 at 4°C overnight. After three washes with 1% BSA, cells were incubated with a FITC conjugated goat anti-mouse IgG polyclonal antibody (KPL Inc., Gaithersburg, MD) at dilution of 1∶20 at room temperature for 1 h. Cells were washed three times with 1% BSA and resuspended in 1× PBS and analyzed by flow cytometry (BD Biosciences) using the CELLQuest™ software (BD Biosciences).

To detect both CHIKV infection and the presence of active caspase 3, the protocol above was followed exactly except that after the 1 h incubation with the secondary antibody, cells were washed twice with 1% BSA and then washed once with BD Perm/Wash buffer (BD Biosciences, San Diego, CA) and cells were subsequently incubated with a phycoerythrin conjugated anti-active caspase 3 antibody according to the manufacturers protocol (BD Biosciences) prior to flow cytometry.

### Protein extraction for 2D-PAGE

Mock and CHIKV infected cells were scraped into 2 ml of serum free medium and centrifuged at 1,800 rpm for 5 minutes. Cells were then resuspended in 1 ml of 1× PBS and centrifuged again at 1,800 rpm for 5 minutes and cell pellets were resuspended in 500 µl ice-cold lysis buffer (7 M urea, 2 M thiourea ,4% w/v CHAPS, and 100 mM dithiothreitol (DTT) containing 1% protease inhibitor cocktail (Bio Basic Inc., Markham, Ontario, Canada). After cell lysis, samples were sonicated at 4°C for 5 minutes 4 times and centrifuged at 19,400× g for 25 minutes at 4°C. The supernatants were then transferred in to new tubes and kept at −80°C until required. Protein concentrations were determined using the Bradford protein assay.

### 2D-PAGE

A total of 250 µg of protein was added to the rehydration buffer (7 M urea, 2 M thiourea, 4% w/v CHAPS, 97 mM dithiothreitol (DTT), 2%v/v IPG buffer (pH3-10, non-linear), 1% bromophenol blue and protease inhibitor cocktail to a final volume of 130.5 µl. The mixture was then centrifuged at 12,000 rpm for 5 minutes and loaded to 7 cm Ettan IPGphor strip holder. The 7 cm, non-linear pH 3–10, Immobilized pH gradient (IPG, GE Healthcare, Buckinghamshire, UK) strips were allowed to rehydrate at 20°C for 16 hours applying 50 µA constant current per IPG strip using Ettan IPGphor system (GE Healthcare). After isoelectric focusing (IEF; step 1, 300 v, 1200 vh; step 2, 1000 v, 300 vh, step 3, 3000 v, 4000 vh; step 4, 5000 v, 4500 vh; step 5, 5000 v, 3000 vh and step 6 100 v, 10.00 hr), the IPG strips were reduced in 50 mM Tris–HCl (pH 8.8), 6 M urea, 30% v/v glycerol, 2% SDS w/v, 50 mg DTT and 1% bromophenol blue for 20 min and then alkylated in the same buffer containing 170 mM iodoacetamide (IAA) for 45 minutes. The equilibrated gel strips were then placed onto 8 mm×8 mm 12.5% sodium dodecyl sulphate polyacrylamide gels (SDS-PAGE) and run at 20 mA/gel constant current for the second dimension separation. After separation, the gels were stained with colloidal Coomassie Brilliant Blue G250 in 40% methanol for 48 hrs and destained with milliQ water for 48 hours, scanned and analyzed with ImageMaster™ 2D Platnium software version 4.9 (GE Healthcare). Manual editing and removal of artifacts was undertaken after automatic spot detection at default parameters. Samples were analyzed in 6 biological replications for each condition.

### Protein extraction for GeLC-MS/MS

Triplicates of CHME-5 cells grown in 60 mm cell culture dish and either mock infected or infected with CHIKV at MOI of 2.5 were collected at 2 d.p.i. by scraping and cells were pelleted by centrifugation at 500× g. After washing with 1× PBS, the cell pellet was resuspended in MilliQ water and 2 volume of ice-cold 100% acetone was added and samples were stored at −30°C until required. The frozen cells were subsequently thawed and centrifuged at 9,200× g for 10 min. The protein pellets were dried in a fume hood for 30 min and samples were resuspended in 0.5% SDS in water. Protein concentration was measured using the Lowry protein assay and 50 µg of protein/lane from each sample was loaded and separated on a 12.5% SDS-PAGE gel. The gel was then stained with colloidal Coomassie blue G and each lane cut into 1×1×1 mm^3^ sized gel plugs.

### In-gel digestion

The gel plugs were placed in wells of a 96 well plate and washed with 200 µl/well of sterile milliQ water by shaking for 5 min at room temperature followed by destaining with 25 mM NH_4_HCO_3_ in 50% methanol until the gel plugs were clear. Destaining solution was removed by washing three times with sterile milliQ water and samples were dehydrated by the addition of 200 µl/well of 100% acetonitrile (ACN) and shaking for 5 min at room temperature and samples were allowed to dry at room temperature for 10 min. The proteins were then reduced by the addition of 20 µl/well of 10 mM dithiothreitol (DTT) in 10 mM ammonium bicarbonate (NH_4_HCO_3_) and incubation at 56°C for 1 hr and then alkylated by the addition of 20 µl/well of 100 mM IAA in 10 mM NH_4_HCO_3_ and further incubation at room temperature in the dark for 1 h followed by washing twice with 100% ACN. The proteins were digested by the addition of 20 ng/well of trypsin in 10 mM NH_4_HCO_3_ and incubation at room temperature for 20 min followed by incubation at 37°C for 3 hrs. The solutions were then transferred to a new low binding 96 well plate. The remaining peptides in the original plate were then extracted twice by the addition of 30 µl/well of 50% ACN in 0.1% formic acid (FA) followed by shaking at room temperature for 10 min and the solutions added to the original digestion solution. The final extracted solution was then incubated at 40°C until dry and samples were kept at −80°C until analysis by LC-MS/MS.

### LC-MS/MS

The dried samples were first dissolved in 10–12 µl/well of 0.1% FA in LC-MS grade water and transferred to the vial for injection. Nanoscale LC separation of tryptic peptides was performed with an Ultimate 3000 LC System (Dionex, USA) coupled to ESI-Ion Trap MS (HCT Ultra PTM Discovery System (Bruker, Germany)) with electrospray at a flow rate of 300 nl/min to a nanocolumn (Onyx monolithic HDC18, 0.2 mm i.d. ×150 mm). Mobile phase A was 0.1% formic acid in water, and mobile phase B was 80% acetonitrile with 0.1% formic acid. A multistep gradient was used to elute peptides: a linear increase from 10%–70% B for 13 min, 90% B at 13–15 min followed by a decrease to 10%B at 15–20 min. Peptide fragment mass spectra were acquired in data-dependent AutoMS mode with a scan range of 300−1500 *m*/*z*, 3 averages, and up to 5 precursor ions selected from the MS scan 50−3000 *m*/*z*.

### Protein quantitation and identification

For protein quantitation, DeCyder MS Differential Analysis software (DeCyderMS, GE Healthcare [37], [38]) was used. Acquired LC-MS raw data were converted and the PepDetect module was used for automated peptide detection, charge state assignments, and quantitation based on the peptide ions signal intensities in MS mode. The analyzed MS/MS data from DeCyderMS were submitted for a database search using the Mascot software (Matrix Science, London, UK, [39]). The data was searched against the NCBI database for protein identification. Database interrogation was; taxonomy (Human); enzyme (trypsin); variable modifications (carbamidomethyl, oxidation of methionine residues); mass values (monoisotopic); protein mass (unrestricted); peptide mass tolerance (1.2 Da); fragment mass tolerance (±0.6 Da), peptide charge state (1+, 2+ and 3+) and max missed cleavages (3). Proteins considered as identified proteins had at least one peptide with an individual mascot score corresponding to p<0.05.

### Western blot analysis

Mock and CHIKV infected cells were collected as described before and resuspended in 50 µl/10^6^ cells of RIPA lysis buffer (1% Nonidate P-40, 0.5% sodium deoxycholate and 0.1% SDS in 1× PBS and PIC added) followed by four rounds of sonication, each for round for 4 min. The cell lysates were then centrifuged at 10,000×g for 15 min and the supernatant kept as a protein stock. 30 µg of total proteins were separated on 10% SDS-PAGE gels and transferred onto 0.2 µm nitrocellulose membranes (Whatman GmbH, Germany) using a constant 30 volts overnight. The membranes were then blocked with 5% skim milk for an hour and probed with an appropriate primary antibody in 5% skim milk for another hour. After incubation with an appropriate secondary antibody conjugated with horseradish peroxidase (HRP) for an hour, the signals were developed using the ECL Prime western blot detection reagent (RPN 2232, GE Healthcare) and detected using autoradiography film. Antibodies used included a 1∶400 dilution of a rabbit anti-JAK 2 polyclonal antibody (sc-278; Santa Cruz Biotechnology Inc.), a 1∶6,000 dilution of a rabbit anti-Hsp90 polyclonal antibody (sc-7947; Santa Cruz Biotechnology Inc.), a 1∶4,000 dilution of a rabbit anti-Hsp70 polyclonal antibody (sc-1060; Santa Cruz Biotechnology Inc.), a 1∶5,000 dilution of a rabbit anti-nucleolin polyclonal antibody (sc-13057; Santa Cruz Biotechnology Inc.), a 1∶3,000 dilution of a rabbit anti-heterogeneous nuclear ribonucleoprotein polyclonal antibody (ab65049; Abcam, Cambridge, MA), a 1∶10,000 dilution of a rabbit anti-elongation factor 2 polyclonal antibody (ab33523; Abcam) all followed by a 1∶4,000 dilution of a goat horseradish peroxidase (HRP) conjugated anti-rabbit IgG polyclonal antibody (31460; Pierce, Rockford, IL) as well as a 1∶5,000 dilution of a goat anti-actin polyclonal antibody (sc-1616; Santa Cruz Biotechnology Inc.) followed by a 1∶8,000 dilution of a rabbit HRP conjugated anti-goat IgG polyclonal antibody (31402; Pierce), as well as a 1∶1000 dilution of a mouse anti-alphavirus monoclonal antibody (Santa Cruz Biotechnology Inc., Santa Cruz, CA) followed by a 1∶5,000 dilution of a rabbit HRP conjugated anti-mouse IgG polyclonal antibody (A9044; Sigma, Sigma-Aldrich, St Louis, MO).

### Semi-quantitative RT-PCR

Total RNA was extracted from infected and mock control CHME-5 cells by using TRI reagent® (Molecular Research Center, Inc., Cincinnati, OH). cDNAs were synthesized from 1.0 µg of total RNA using oligo (dT) primer (Bio Basic, Inc.) and ImpromII™ reverse transcriptase (Promega, Madison, WI). Specific primers for each gene were used to amplify the synthesized cDNA by PCR and primer sequences, cycle conditions and expected product sizes are given in Supplementary Table S1. The PCR products were then separated on 2% agarose gel and products visualized by staining with ethidium bromide.

## Results

### 2D-PAGE analysis of CHIKV infected CHME-5 cells

**Figure 1:**
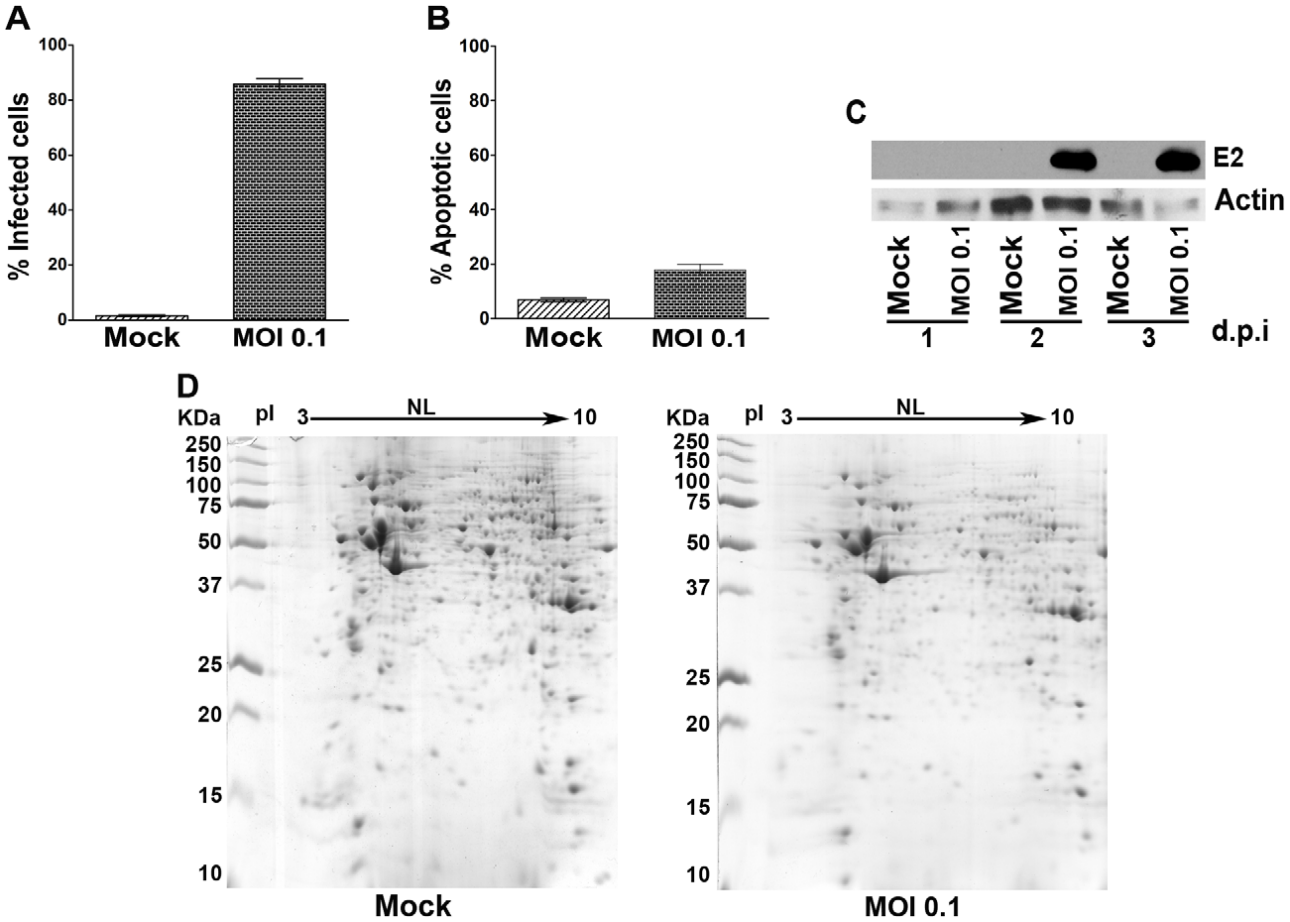
Analysis of CHIKV infected CHME-5 cells. CHME-5 cells either mock infected or infected with CHIKV at MOI 0.1 were collected at day 2 p.i. (A, B, D) or on days 1 to 3 p.i. (C) and subsequently (A) stained with an anti-alphavirus antibody and the percentage of infected cells analyzed by flow cytometry or (B) stained with Annexin V-FITC and PI and the percentage of apoptotic cells analyzed by flow cytometery or (C, D) used for total protein extraction and (C) analyzed by western blotting with an anti-alphavirus monoclonal antibody and an anti-actin polyclonal antibody or (D) the differential proteome determined by 2D-PAGE. Representative gels from 6 biological replicates are shown. (A and B) Bar graphs represent the means ± SD of 6 replications.

To confirm the susceptibility of CHME-5 cells to CHIKV, cells were infected at MOI 0.1 and on day 2 p.i. fixed, permeabilized and stained with a monoclonal anti-alphavirus antibody followed by an appropriate FITC-conjugated secondary antibody before being analyzed in a flow cytometer. Results (Figure 1A) showed that slightly more than 80% of cells were infected at this time point. Under this protocol, less than 20% of cells were undergoing apoptosis as assessed by FITC conjugated Annexin V/propidium iodide staining (Figure 1B). These results are compatible with our previous study undertaken at MOI 1 [30]. While the percentage of apoptotic cells in this study is markedly lower than that reported in a previous study on CHIKV infected HeLa cells [2], that study was undertaken at MOI 10. We further established that infection was coupled with the production of viral proteins by infecting CHME-5 cells with CHIKV at MOI 0.1 and analyzing the expression of CHIKV proteins via western blot analysis using an anti-alphavirus monoclonal antibody in parallel with mock infected cells (Figure 1C) which showed robust CHIKV protein expression on day 2 p.i. which is consistent with our previous data showing CHIKV protein expression in infected CHME-5 cells by immunocytochemistry [30].

To provide an initial analysis of the proteomic changes in CHME-5 cells in response to CHIKV infection, cells were infected or mock infected with CHIKV at MOI of 0.1 and on day 2 p.i. cells were lysed and the harvested proteins subjected to 2D analysis in parallel with mock infected cells. Experiment was undertaken as six independent replicates. Results (Figure 1D) showed that a total of 59 protein spots were differentially expressed (at p<0.05) in response to CHIKV infection, with 58 of these spots being down regulated (with 13 spots being found only in the mock infected samples) while 1 spot was up-regulated in the infected cells. Given the apparent large number of proteins differentially regulated in response to CHIKV analysis, further analysis was conducted by GeLC-MS/MS.

### GeLC-MS/MS Analysis

**Figure 2:**
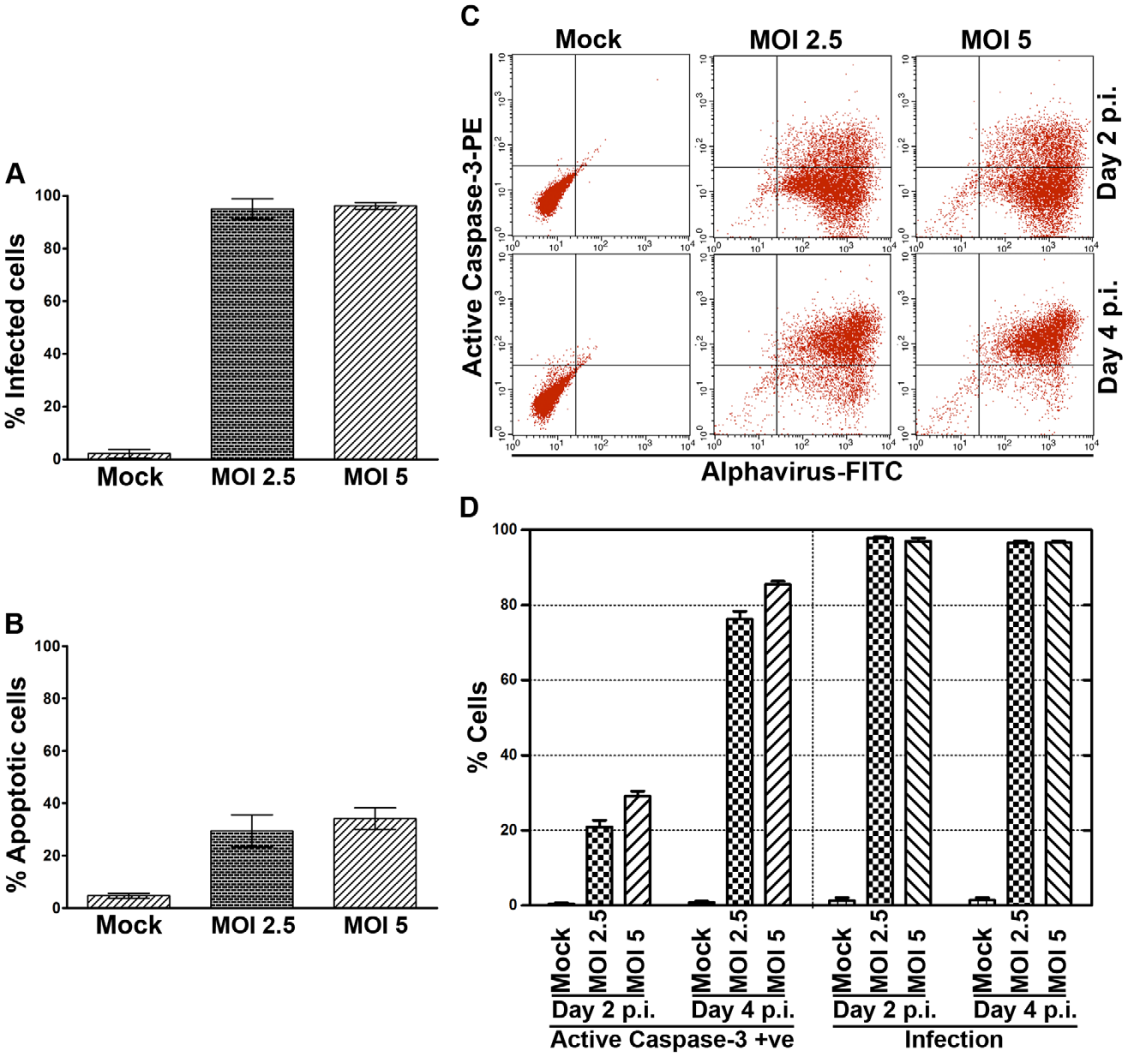
Infection and apoptosis in CHIKV infected CHME-5 cells. (A and B) CHME-5 cells either mock infected or infected with CHIKV at MOI 2.5 or 5 were collected at day 2 p.i. and (A) cells were stained with an anti-alphavirus antibody and the percentage of infected cells was determined by flow cytometry or (B) cells were stained with Annexin V-FITC and PI and the percentage of induced apoptosis determined by flow cytometery. Bar graphs represent the means ± SD of 3 replications per group. (C and D) CHME-5 cells either mock infected or infected with CHIKV at MOI 2.5 or 5 were collected on days 2 and 4 p.i. and analyzed by flow cytometry after double staining with antibodies directed against active caspase 3 and alphavirus. Experiment was undertaken in three independent replicates. Representative flow cytometry dot plot is shown in (C) and data is shown graphically in (D). Bar graphs represent the means ± SD of 3 replications per group.

Before undertaking the GeLC-MS/MS analysis, the level of infection was re-optimized to ensure nearly all cells were infected. Cells were infected at MOI 2.5 and 5 as previously, and cells were analyzed for the levels of infection and induction of apoptosis by flow cytometry as previously. Results showed that both MOI 2.5 and 5 gave nearly 100% infection (Figure 2A), albeit with a slight increase in the number of cells undergoing apoptosis (Figure 2B). To confirm that the cells undergoing apoptosis were CHIKV infected cells, cells were infected at MOI 2.5 and 5 as previously, and cells were analyzed on days 2 and 4 p.i. by double staining with antibodies directed against active caspase 3 and against alphaviruses. Results (Figure 2C, D) showed that the percentage of active caspase 3 positive cells on day 2 was in close agreement with the percentage of Annexin V/propidium iodide positive cells as seen in the earlier analysis (Figure 2B) for both MOI 2.5 and 5 and moreover that the active caspase 3 positive cells correlated with alphavirus positive cells over the period of the experiment (Figure 2C).

CHME-5 cells were therefore mock infected or infected with CHIKV at MOI 2.5 and on day 2 p.i. proteins were harvested and subjected to GeLC-MS/MS analysis. Experiment was undertaken as three independent replicates.

**Figure 3:**
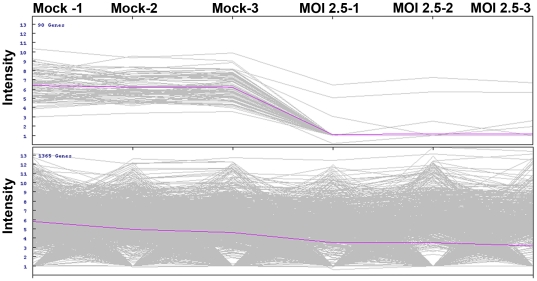
GeLC-MS/MS analysis of the proteome of CHIKV infected CHME-5 cells. CHME-5 cells either mock infected or infected with CHIKV at MOI 2.5 were collected at day 2 p.i. and proteins extracted and the proteomes determined by GeLC-MS/MS. Each line of the graph represents a single protein and the intensity of individual proteins is shown. The upper panel of the graph shows differentially expressed proteins significant at *p*<0.01. The lower panel shows proteins which are not significant at *p*<0.01. Samples were analyzed as three independent replicates.

**Figure 4:**
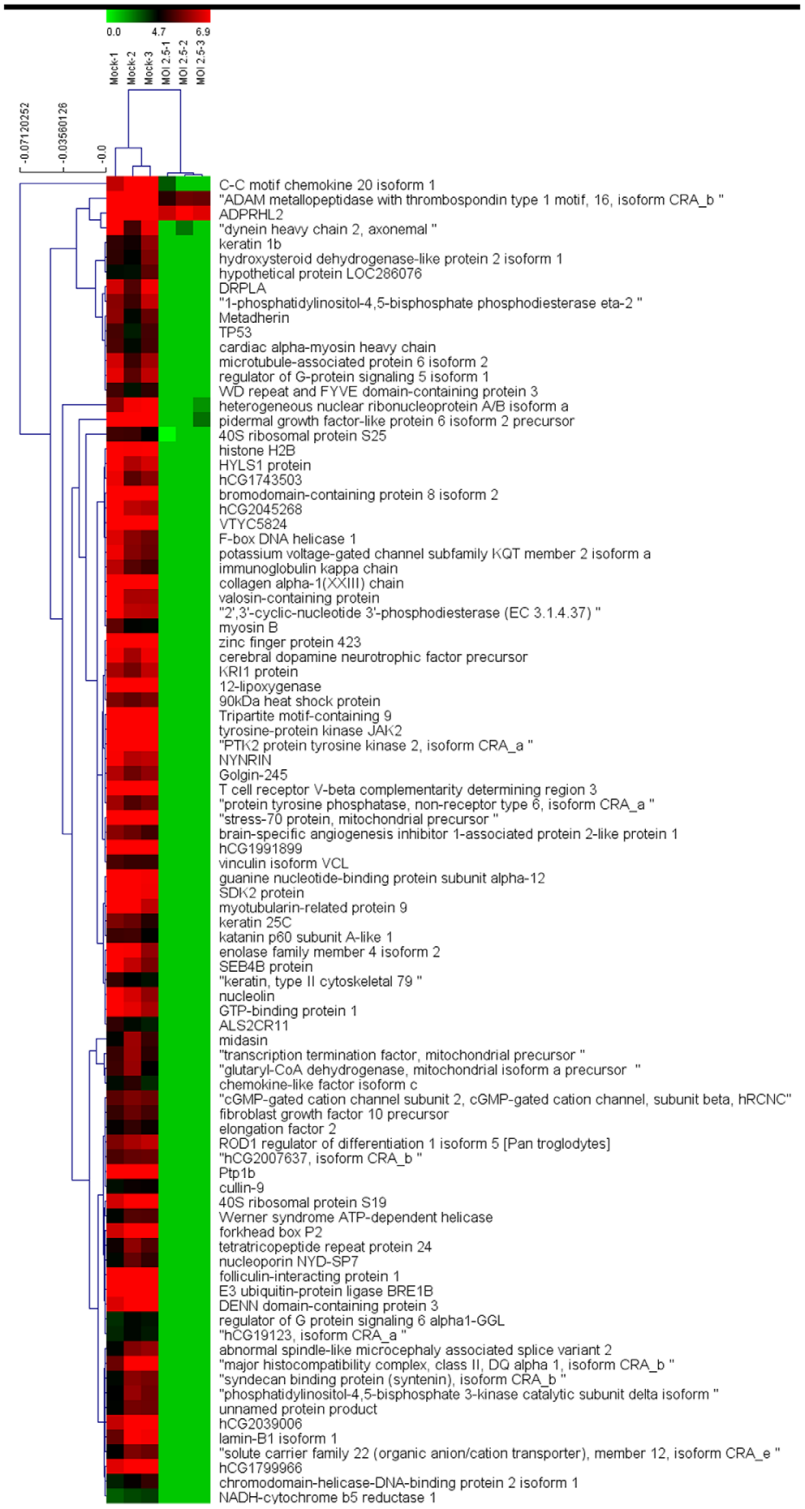
Two-way hierarchical clustering analysis of 90 significantly differentially expressed proteins in mock (left) and CHIKV (right) infected CHME-5 cells. Each column represents a single biological replication and each line represents a single protein. The color scale is shown by the bar at the top.

**Figure 5:**
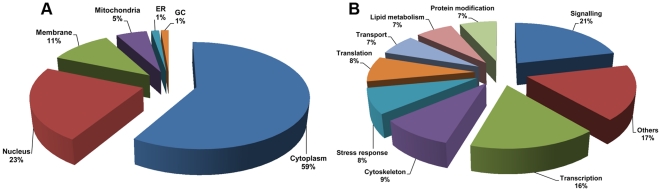
Sub-cellular distribution and functional annotation of proteins significantly differentially expressed in response to CHIKV infection of CHME-5 cells. The sub-cellular (A) and functional (B) categorization of the proteins was performed using the GoCat software.

GeLC-MS/MS analysis revealed a total of 1455 differentially expressed proteins, of which 90 proteins were differentially expressed at a significance level of p<0.01 (Figure 3). Two-way hierarchical clustering analysis (Figure 4) of these differentially expressed proteins shows almost all of the differentially expressed proteins were down regulated in infected cells, which is consistent with the 2D-PAGE result. A full list of the 90 differentially expressed proteins is given in Supplementary Table S2, and the cellular distribution and a functional annotation of the 90 highly significantly differentially expressed proteins is given in Figure 5.

### Validation of GeLC-MS/MS results

**Figure 6:**
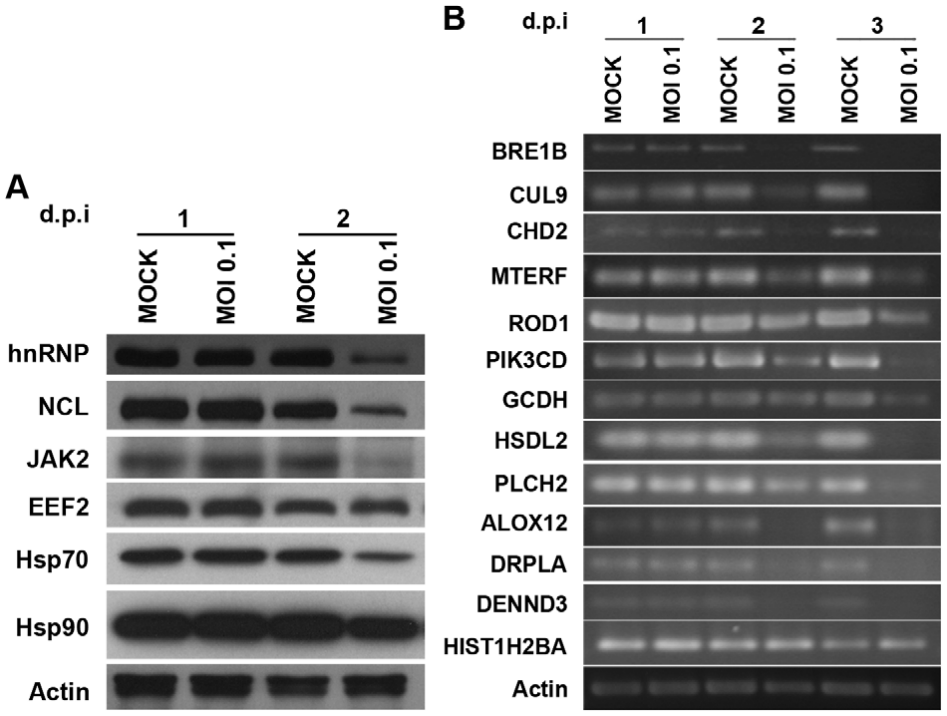
Validation of proteins differentially expressed in response to CHIKV infection in CHME-5 cells. (A) CHME-5 cells were either mock infected or infected with CHIKV at MOI 0.1 before extraction of proteins and analysis by Western blot analysis on 1 and 2 d.p.i. hnRNP: heterogeneous nuclear ribonucleoprotein; NCL: nucleolin; JAK2: tyrosine-protein kinase JAK2; Hsp70: heat shock protein 70; Hsp90: heat shock protein 90. (B). CHME-5 cells were either mock infected or infected with CHIKV at MOI 0.1 before extraction of total RNA and analysis by RT-PCR on 1, 2 and 3 d.p.i. BRE1B: E3 ubiquitin-protein ligase; CUL9: Cullin-9; CHD2: chromodomain-helicase-DNA binding protein 2; MTERF: mitochondrial precursor transcription termination factor; ROD1: regulator of differentiation 1 isoform; PIK3CD: phosphatidylinositol-4,5-bisphosphate 3-kinase catalytic subunit delta; GCDH: mitochondrial glutaryl-CoA dehydrogenase isoform precursor; HSDL2: hydroxysteroid dehydrogenase-like protein 2; PLCH2: 1-phosphatidylinositol-4,5-bisphosphate phosphodiesterase eta-2; ALOX12: 12-lipoxygenase; DRPLA: Dentatorubral pallidoluysian atrophy protein; DENND3: DENN domain-containing protein 3; HIS1H2B: Histone 2B.

A total of 6 of the highly differentially expressed proteins, namely heterogeneous nuclear ribonucleoprotein (hnRNP), nucleolin (NCL), tyrosine-protein kinase JAK2 (JAK2), eukaryotic elongation factor 2 (EEF2), heat shock protein 70 (Hsp70) and heat shock protein 90 (Hsp90) together with actin were selected for confirmatory Western analysis. The candidates were selected as being representative of signaling (JAK2), stress response (Hsp70 and Hsp90) transcription (NCL and hnRNP) and translation (EEF2) pathways and results (Figure 6A) were consistent with the GeLC-MS/MS analysis with all proteins showing significant down regulation by day 2 p.i.. A further 13 proteins, namely E3 ubiquitin-protein ligase (BRE1B), Cullin-9 (CUL9), chromodomain-helicase-DNA binding protein 2 (CHD2), mitochondrial precursor transcription termination factor (MTERF), regulator of differentiation 1 isoform (ROD1), phosphatidylinositol-4,5-bisphosphate 3-kinase catalytic subunit delta (PIK3CD), mitochondrial glutaryl-CoA dehydrogenase isoform precursor (GCDH), hydroxysteroid dehydrogenase-like protein 2 (HSDL2), 1-phosphatidylinositol-4,5-bisphosphate phosphodiesterase eta-2 (PLCH2), 12-lipoxygenase (ALOX12), Dentatorubral pallidoluysian atrophy protein (DRPLA), DENN domain-containing protein 3 (DENNDD3) and Histone 2B (HIST1H2B) together with actin were selected for analysis by semi-quantitative RT-PCR analysis to evaluate expression at the level of the RNA message, with the selected candidates representative of the apoptosis (DENNDD3 and DRPLA), lipid metabolism (ALOX12, PLCH2, HSDL2, GCDH, PIK3CD), transcription (ROD1, MTERF, HIST1H2B and CHD2) and ubiquitinylation (BRE1B and CUL9). Results (Figure 6B) showed that almost all of the selected candidates were down regulated at the level of gene expression (transcription) with the exception of Histone H2B which was found up regulated at the level of transcription.

## Discussion

Despite intensive study over the last few years, the detailed mechanism of the pathology of CHIKV infection remains to be established. Both old world and new world alphaviruses have been reported to induce both transcriptional and translational shut off in infected cells and although the mechanism of this is not completely known, both the nsP2 protein and the capsid protein have been implicated in the process of transcriptional shut off [32], [33]. SINV has been shown to induce PKR dependant and PKR independent translational shut off [40], and while CHIKV infection induces PKR mediated phosphorylation of eIF2α, it is not essential to the process of global translational shut off, suggesting that alternate, PKR independent mechanisms exist, which are possibly specifically targeted at the host cell antiviral response [34]. In this regard, the cell line used in this study, CHME-5, was derived from primary human embryonic microglia by transfection with SV40 large T antigen [36], which has been shown to rescue translational arrest induced by PKR mediated phosphorylation of eIF2α [41]. While this could have possibly confounded the results generated, as noted, PKR mediated phosphorylation of eIF2α is not believed to be essential to the process of global translational arrest in CHIKV infection [34].

One possible explanation for the results seen in our proteomic analysis is simply that after global translational arrest, proteins that are shown as strongly down regulated are simply proteins with short half-lives which are removed from the cell by normal protein turn over by various cellular processes [42]. We therefore looked at the established half-lives of a number of proteins to determine whether the strongly down regulated proteins were associated with short half lives, while the proteins that were not significantly down regulated had long half lives. Protein half-lives were sourced from a number of publications [43], [44], [45]. As shown in Table 1, there was no discernable association between protein half life and down regulation significance or lack of it in the proteomic study. This was exemplified by histone H2B which has a comparatively long half life of 30 hours [45], but was strongly down regulated in response to CHIKV infection. Similarly the 40S ribosomal protein S8 which has a half-life of 3 hours [43] was found at essentially similar levels in mock and CHIKV infected cells. The time of sample analysis was 48 hours post infection which would represent up to 16 half lives for the 40S ribosomal protein S8 which would result in the effective complete disappearance of this protein. Therefore the proteomic results do not appear consistent with a simple global translational shut off.

**Table 1:** Values of fold change in response to CHIKV infection, significance and half life of selected proteins.

Protein	Fold change	p-value	Half-life(hrs)
elongation factor 2	4.91	<0.001	8.8
Heat shock protein 90 KDa alpha	5.69	<0.001	4.2
stress-70 protein, mitochondrial precursor	7.35	0.001	10.6
Nucleolin	6.74	0.008	13.9
Vinculin isoform VCL	5.24	<0.001	11.7
Histone H2B	7.78	0.009	30
TP53	4.94	0.009	0.2
myosin-9	0.96	NS	6.4
proteasome subunit alpha type-6	1.9	NS	8.5
proteasome subunit alpha type-7	1.2	NS	9
ribosomal protein S2	0.9	NS	19.7
40S ribosomal protein S8	0.7	NS	3.0
60S ribosomal protein L22 proprotein	1.2	NS	11.1
Vimentin	1.0	NS	4.9

In cases where eIF2α mediated translational inhibition occurs, translation can still occur through the utilization of the internal ribosome entry site (IRES) whereby the ribosome is directly recruited to a site within the 5′-UTR of the mRNA [46]. IRES directed translation initiation commonly occurs under conditions where normal, cap-dependant translation is reduced [47]. While there are few well characterized cellular mRNAs that are capable of IRES-mediated translation, proteins capable of IRES mediated translation including p53 [48] and Hsp70 [49] were found in the significantly down regulated group, suggesting that the result we observed does not arise from IRES-mediated translation generating the bulk of the proteins showing no significant change in expression. Collectively these results suggest that there is a degree of targeted protein down regulation in addition to any more global processes ongoing. Furthermore while the Western analysis confirmed that all proteins we validated were down regulated at the level of protein expression, at least one gene (Histone 2B) was found to be transcriptionally up-regulated (as evidenced by semi-quantitative RT-PCR), but translationally down-regulated (as evidenced in the GeLC-MS/MS analysis), suggesting that targeted transcriptional inhibition may also play a role in CHIKV infection.

Nearly one quarter of all significantly down regulated proteins were involved with cell signal transduction processes. These included the G-Protein signaling pathway (GTP-binding protein 1, guanine nucleotide-binding protein subunit alpha-12, regulator of G protein signaling 6 alpha1-GGL and regulator of G-protein signaling 5 isoform 1) and the cytokine receptor JAK/STAT signaling pathway signaling (JAK2). Many viruses including tick borne encephalitis [50] and Sindbis virus [51] are known to inhibit the interferon signaling pathway through different mechanisms to suppress the innate antiviral immune response. Chikungunya virus infection has also been shown to suppress the secretion of IFN-β and translation of interferon stimulated genes (ISGs) in human fibroblast cells [34], most likely through the action of CHIKV nsP2 which inhibits interferon stimulated JAK/STAT signaling through blocking phosphorylation and nuclear translocation [52]. Fros and colleagues showed no decrease in the level of endogenous STAT protein [52], and STAT was not shown to be significantly down regulated in this study. Markedly however, JAK2 was shown to be significantly down regulated in this study suggesting that JAK2, rather than STAT may be the primary target of nsP2 mediated inhibition of interferon stimulated JAK/STAT signaling.

Another major cellular process altered was lipid metabolism (7% of significantly down regulated proteins) and viral infections including dengue virus [53], [54], [55] West Nile virus [56] and hepatitis virus [57] are known to induce changes in the lipid metabolism pathway of the host cell. However, these viruses normally either up-regulate lipid biosynthesis or co-opt cholesterol biosynthesis for their own production. While Blanc and colleagues recently showed that the viral induced innate immune response, signaling through the interferon receptor, could down-regulated the sterol biosynthesis pathway in response to infection in macrophages [58], the evidence presented here suggests that the innate immunity pathway is down regulated in response to CHIKV infection. This suggests either a temporal difference in that innate immunity triggers down regulation of lipid metabolism prior to the inhibition of this pathway, or down regulation of lipid biosynthesis occurs as a result of transcriptional or translational shut off. Another protein in the stress response group, the p53 tumour suppressor protein, has also been shown to be involved in interferon mediated antiviral defense acting through the JAK/STAT signaling cascade [59], suggesting that down regulation of the interferon response pathway is a major target of the CHIKV transcriptional or translational shut off.

In total, 6 (8%) of the proteins identified as highly down regulated in response to CHIKV infection were classified in the stress response group which included both Hsp70 and Hsp90 as well as p53 as noted above. Heat shock proteins are multifunctional proteins with important roles in folding and assembly of newly synthesized proteins, the refolding of aggregated or mis-folded proteins as well as protein trafficking and the regulation of signal transduction [60]. Hsp70 has been shown to block replication of Influenza A virus possibly through disrupting the polymerase-viral RNA complex binding [61] and while a role for Hsp90 in this inhibition has not been proposed yet, Hsp90 and Hsp70 are frequently complexed through the action of Hop or Hsp70/Hsp90 Organizing Protein [62].

A recent proteomic study using CHIKV infected suckling mice identified 35 differentially down regulated proteins in liver and 15 differentially down regulated proteins in brain, which primarily belonged to stress, inflammation, apoptosis, urea cycle and energy metabolism pathways [63]. Both our study and the study of Dhanwani [63] detect changes in the apoptosis, stress response and lipid metabolism pathways suggesting the importance of these pathways, albeit that largely different proteins in these pathways were identified by the two studies. However, there are several important technical differences between the Dhanwani study [63] and our study. In particular the lower number of proteins detected by Dhanwani and colleagues [63], even when compared to our 2D-PAGE analysis, where we detected 59 differentially regulated spots may result from the use of tissues of a mixed origin. For example liver specimens may consist of hepatocytes, liver sinusoidal cells, kuppfer cells and other cell types as well as supporting and vascular cells [64]. In this case the sample may represent a mixed population of cells, of which many may not be infected, serving to dilute the sensitivity of the analysis. Our analysis, using a single cell type at a high level of infection, particularly when coupled to the more sensitive analysis of GeLC-MS-MS, should provide a more detailed analysis, as seen by the larger number of proteins identified as being down regulated in response to infection.

In conclusion, this study shows that CHIKV infection of CHME-5 cells caused the down regulation of proteins in different interrelated cellular pathways including cell signaling, lipid metabolism, protein modification, transcription, translation, and stress responses. Many of the differentially expressed proteins from the different pathways however are related to the antiviral innate immune response of the host cell, suggesting that specific down regulation of host cell antiviral responses is one of the primary targets of the apparent global transcriptional shut off.

## Supporting Information

Table S1 **List of primers and cycle conditions used in RT-PCR analysis.**
(DOC)

Table S2 **List of proteins significantly differentially expressed in CHME 5 cells in response to CHIKV infection.**
(DOC)
