# Supplementary material for: Proteomic Analysis of Chikungunya Virus Infected Microgial Cells
Source: PLoS One. 2012 Apr 13;7(4):e34800. doi: 10.1371/journal.pone.0034800 (PMC3326055; doi:10.1371/journal.pone.0034800)
Supplement: Table S1 — List of primers and cycle conditions used in RT-PCR analysis. (DOC) [file pone.0034800.s001.doc]

**Supplementary Table S1**. List of primers and cycle conditions used in RT-PCR analysis.

| Primer name | Start | Sequence | PCR condition | Product size |
| --- | --- | --- | --- | --- |
| DENND3:_ F DENND3:_ R | 364  474 | 5’-CGGGCTGGGTATTAAAGACA-3’  5’-CTCAAGGACTGCCTTTCCTG-3’ | 94 ^o^c, 20 sec; 55 ^o^c, 45 sec; 72 ^o^c, 45 sec; 30 cycles | 111 |
| DRPLA:_ F  DRPLA:_ R | 2608  2857 | 5’-TACCTGGGTCCTGACACTCC-3’  5’-CCAGCTCACTAGGCTTCACC-3’ | 94 ^o^c, 20 sec; 55 ^o^c, 30 sec; 72 ^o^c, 45 sec; 20 cycles | 250 |
| ALOX12:_ F  ALOX12:_ R | 692  991 | 5’-AGTTCCTCAATGGTGCCAAC-3’  5’-ACAGTGTTGGGGTTGGAGAG-3’ | 94 ^o^c, 20 sec; 55 ^o^c, 30 sec; 72 ^o^c, 45 sec; 30 cycles | 300 |
| PLCH2:_ F  PLCH2:_ R | 1910  1666 | 5’-GCTGAAGAGGACGTGGAGTC-3’  5’-GCCACGGACTTGGTGTACTT-3’ | 94 ^o^c, 20 sec; 55 ^o^c, 30 sec; 72 ^o^c, 45 sec; 30 cycles | 245 |
| HCDL2:_ F  HCDL2:_ R | 906  1027 | 5’-ACCAAAACCACGTTCTGGAG-3’  5’-CACCATCTTCACCGGAGAGT-3’ | 94 ^o^c, 20 sec; 55 ^o^c, 30 sec; 72 ^o^c, 45 sec; 30 cycles | 122 |
| GCDH:_ F  GCDH:_ R | 396  625 | 5’-GGTGGACAGTGGCTACAGGT-3’  5’-TCCCATTGAGGGTGTAGCTC-3’ | 94 ^o^c, 20 sec; 55 ^o^c, 45 sec; 72 ^o^c, 45 sec; 30 cycles | 230 |
| PIK3CD:_ F  PIK3CD:_ R | 12131598 | 5’-CGCTCCACCAAGAAGAAGTC-3’  5’-TTCCACACCAGGTCCTTCTC-3’ | 94 ^o^c, 20 sec; 55 ^o^c, 30 sec; 72 ^o^c, 45 sec; 30 cycles | 386 |
| ROD1:_ F  ROD1:_ R | 10591308 | 5’-CATTCCTGGGGCTAGTGGTA-3’  5’-GGACAGTGTAGCACGAAGCA-3’ | 94 ^o^c, 20 sec; 55 ^o^c, 30 sec; 72 ^o^c, 45 sec; 30 cycles | 250 |
| MTERF:_ F  MTERF:_ R | 479  659 | 5’-TGGAACGTTCTCCTGAATCC-3’  5’-GCCTGCAAAAATTCAACCAT-3’ | 94 ^o^c, 20 sec; 55 ^o^c, 30 sec; 72 ^o^c, 45 sec; 25 cycles | 181 |
| CHD2:_ F  CHD2:_ R | 1734  2234 | 5’-ATGGATTCATTCCCAAACCA-3’  5’-CTTGTTCCTTTGGCAAGAGC-3’ | 94 ^o^c, 20 sec; 55 ^o^c, 30 sec; 72 ^o^c, 45 sec; 25 cycles | 501 |
| HIST1H2BA:_F  HIST1H2BA:_F | 57  267 | 5’-GACCAAAGCGCAGAAGAAAG-3’  5’-GGTCGAGCGCTTGTTGTAAT-3’ | 94 ^o^c, 20 sec; 55 ^o^c, 30 sec; 72 ^o^c, 45 sec; 25 cycles | 211 |
| CUL9:_ F  CUL9:_ R | 46454829 | 5’-TTTGCCAGGTACATTGACCA-3’  5’-GAACCAAAGCTCAGGAGACG-3’ | 94 ^o^c, 20 sec; 55 ^o^c, 30 sec; 72 ^o^c, 45 sec; 25 cycle | 185 |
| BRE1B:_ F  BRE1B:_ R | 2907  2277 | 5’-TGTGACAGGTCAGGCTTTTG-3’  5’-GAAGCAGAAAACGTGGAAGC-3’ | 94 ^o^c, 20 sec; 55 ^o^c, 30 sec; 72 ^o^c, 45 sec; 25 cycle | 631 |
| Actin:_ F  Actin:_ R |  | 5’-GAAGATGACCCCAGATCATGT-3’  5’-ATCTCTTGCTCGAAGTCCAG-3’ | 94 ^o^c, 20 sec; 55 ^o^c, 30 sec; 72 ^o^c, 45 sec; 20 cycle | 330 |
