# Supplementary material for: Proteomic Analysis of Chikungunya Virus Infected Microgial Cells
Source: PLoS One. 2012 Apr 13;7(4):e34800. doi: 10.1371/journal.pone.0034800 (PMC3326055; doi:10.1371/journal.pone.0034800)
Supplement: Table S2 — List of proteins significantly differentially expressed in CHME 5 cells in response to CHIKV infection. (DOC) [file pone.0034800.s002.doc]

Supplementary Table S2: List of proteins significantly differentially expressed in CHME 5 cells in response to CHIKV infection.

| **Protein name** | **Accession number** | **Peptide sequence** | **Mowse score*** | **MH+*** | **Mock mean*** | **Infect mean*** | **Fold change*** | **Adj p value**** |
| --- | --- | --- | --- | --- | --- | --- | --- | --- |
| 1-phosphatidylinositol-4,5-bisphosphate phosphodiesterase eta-2 | gi|78499633 | LNVNLPR | 6.85 | 828.92 | 5.85 | 1 | 5.85 | 0.005 |
| 2',3'-cyclic-nucleotide 3'-phosphodiesterase | gi|180687 | LSPTDNLPRGSR | 7.87 | 1312.94 | 6.85 | 1 | 6.85 | 0.008 |
| ADAM metallopeptidase with thrombospondin type 1 motif, 16, isoform CRA_b | gi|119628523 | KCSHLPKPSLELER | 5 | 1694.93 | 8.01 | 5.45 | 1.47 | 0.003 |
| PTK2 protein tyrosine kinase 2, isoform CRA_a | gi|119612616 | LMLPRPQECR | 18.70 | 1300.78 | 7.48 | 1 | 7.48 | 0.003 |
| cGMP-gated cation channel subunit 2, cGMP-gated cation channel, subunit beta, hRCNC2 | gi|765320 | SNNKPKEEK | 13.77 | 1077.62 | 5.63 | 1 | 5.63 | <0.001 |
| dynein heavy chain 2, axonemal | gi|75677365 | TVISPR | 28.79 |  | 6.58 | 1.50 | 4.37 | 0.01 |
| glutaryl-CoA dehydrogenase, mitochondrial isoform a precursor | gi|4503943 | AHYNSSNK | 15.93 | 924.18 | 5.31 | 1 | 5.31 | 0.007 |
| hCG19123, isoform CRA_a | gi|119575887 | HSDHDSGKPSSR | 3.52 | 1304.76 | 4.27 | 1 | 4.27 | 0.003 |
| hCG2007637, isoform CRA_b | gi|119629770 | MSVDCVPLKEK | 16.41 | 1309.86 | 5.52 | 1 | 5.52 | <0.001 |
| keratin, type II cytoskeletal 79 | gi|32567786 | KLLESEESR | 15.49 | 1090.32 | 4.73 | 1 | 4.73 | 0.004 |
| major histocompatibility complex, class II, DQ alpha 1, isoform CRA_b | gi|119570890 | SNSTAATNGEEMLVGR | 9.27 | 1635.49 | 6.74 | 1 | 6.74 | 0.009 |
| phosphatidylinositol-4,5-bisphosphate 3-kinase catalytic subunit delta isoform | gi|156564405 | LINSQISLLIGK | 3.71 | 1301.90 | 5.45 | 1 | 5.45 | 0.008 |
| protein tyrosine phosphatase, non-receptor type 6, isoform CRA_a | gi|119609106 | MAWQENSRVIVMTTR | 2.84 | 1820.21 | 5.74 | 1 | 5.75 | 0.002 |
| solute carrier family 22 (organic anion/cation transporter), member 12, isoform CRA_e | gi|119594681 | LENFSAAVPSHR | 2.14 | 1326.48 | 5.37 | 1 | 5.37 | 0.006 |
| stress-70 protein, mitochondrial precursor | gi|24234688 | DAGQISGLNVLR | 50.46 | 1239.17 | 7.35 | 1 | 7.35 | 0.001 |
| syndecan binding protein (syntenin), isoform CRA_b | gi|119607219 | TITMHKDSTG | 5.33 | 1080.54 | 5.41 | 1 | 5.41 | 0.009 |
| transcription termination factor, mitochondrial precursor | gi|5902010 | KIVTSDLEIVNILER | 4.81 | 1570.26 | 5.43 | 1 | 5.43 | 0.005 |
| 12-lipoxygenase | gi|187171 | DDLPPNMR | 20.03 | 972.75 | 7.53 | 1 | 7.53 | <0.001 |
| 40S ribosomal protein S19 | gi|4506695 | DVNQQEFVR | 37.07 | 959.67 | 7.25 | 1 | 7.25 | 0.003 |
| 40S ribosomal protein S25 | gi|4506707 | AALQELLSK | 35.80 | 969.68 | 5.06 | 0.70 | 7.26 | 0.001 |
| 90kDa heat shock protein | gi|306891 | EQVANSAFVER | 59.88 | 1257.59 | 5.69 | 1 | 5.69 | <0.001 |
| ADPRHL2 | gi|48146591 | FAQEYEK | 9.68 | 914.93 | 9.85 | 6.77 | 1.47 | 0.004 |
| ALS2CR11 | gi|15823651 | GNSSLIKEQK | 9.87 | 1089.69 | 4.59 | 1 | 4.59 | 0.006 |
| C-C motif chemokine 20 isoform 1 | gi|4759076 | FIVGFTR | 8.34 | 838.31 | 7.45 | 1.68 | 4.43 | 0.007 |
| DENN domain-containing protein 3 | gi|50345870 | HMQLGDFMK | 3.75 | 1108.25 | 7.37 | 1 | 7.37 | 0.004 |
| DRPLA | gi|862330 | VAGLGNDPLAR | 11.73 | 1074.14 | 6.25 | 1 | 6.25 | 0.007 |
| E3 ubiquitin-protein ligase BRE1B | gi|7662230 | ELEERDR | 26.48 | 945.28 | 8.73 | 1 | 8.73 | 0.005 |
| F-box DNA helicase 1 | gi|21666261 | QAERVFPSNVICK | 3.64 | 1410.38 | 6.06 | 1 | 6.06 | 0.003 |
| GTP-binding protein 1 | gi|82546879 | RVGDNDFLEVR | 7.49 | 1319.92 | 6.83 | 1 | 6.83 | 0.005 |
| Golgin-245 | gi|39655046 | TSATKGTGLDEAXEWLVETLK | 12.08 | 3758.40 | 5.96 | 1 | 5.96 | 0.001 |
| HYLS1 protein | gi|15929192 | MLAAATAFTHIR | 14.13 | 1317.80 | 6.88 | 1 | 6.88 | 0.007 |
| KRI1 protein | gi|85700330 | QLKESFR | 11.61 | 914.34 | 6.08 | 1 | 6.08 | 0.001 |
| Metadherin | gi|28277147 | TELGLDLGLEPKR | 10.17 | 1439.91 | 5.36 | 1 | 5.36 | 0.009 |
| NADH-cytochrome b5 reductase 1 | gi|49574502 | YLLRLLDK | 15.79 | 1021.75 | 3.30 | 1 | 3.30 | 0.006 |
| NYNRIN | gi|126723547 | ASVSLLK | 13.07 | 716.52 | 6.51 | 1 | 6.51 | 0.001 |
| Ptp1b | gi|6729771 | XMEKEFEQIDK | 4.56 | 1399.88 | 8.05 | 1 | 8.05 | 0.001 |
| ROD1 regulator of differentiation 1 isoform 5 | gi|114626184 | RGSDELLSSGIINGPFTMNSSTPSTANGNDSK | 12.29 | 4231.08 | 6.10 | 1 | 6.10 | 0.001 |
| SDK2 protein | gi|62024162 | NLSRPSLTQYELDNLNK | 7.83 | 2004.59 | 7.94 | 1 | 7.94 | 0.007 |
| SEB4B protein | gi|481241 | FVNVVPTFGK | 28.35 | 1107.72 | 6.37 | 1 | 6.37 | 0.004 |
| T cell receptor V-beta complementarity determining region 3 | gi|1432002 | TTNEQFFGPGTR | 8.09 | 1354.92 | 7.54 | 1 | 7.54 | 0.002 |
| TP53 | gi|187233956 | TIHYNYMCDSSCMGGMNR | 2.46 | 2211.64 | 4.94 | 1 | 4.94 | 0.009 |
| Tripartite motif-containing 9 | gi|39795457 | TEGGITKGATIGVLLDFNR | 5.82 | 1961.16 | 7.80 | 1 | 7.80 | 0.003 |
| VTYC5824 | gi|37181528 | MVTYCIWCCDPTHLPIPAEKPVTMR | 8.77 | 3105.99 | 7.34 | 1 | 7.34 | 0.004 |
| WD repeat and FYVE domain-containing protein 3 | gi|31317272 | QLLIKAVNR | 6.77 | 1063.66 | 4.92 | 1 | 4.92 | 0.005 |
| Werner syndrome ATP-dependent helicase | gi|6136393 | LKENMER | 15.69 | 900.68 | 5.15 | 1 | 5.15 | 0.004 |
| abnormal spindle-like microcephaly associated splice variant 2 | gi|66474491 | ASAIIIQR | 13.90 | 870.57 | 5.48 | 1 | 5.48 | 0.009 |
| brain-specific angiogenesis inhibitor 1-associated protein 2-like protein 1 | gi|32171238 | TTSTFK | 17.46 | 285.25 | 5.60 | 1 | 5.60 | 0.002 |
| bromodomain-containing protein 8 isoform 2 | gi|34452707 | MIASHR | 15.98 | 703.44 | 7.94 | 1 | 7.94 | 0.009 |
| cardiac alpha-myosin heavy chain | gi|297024 | TDAQMADFGAAAQYLR | 5.95 | 1734.99 | 5.07 | 1 | 5.07 | 0.004 |
| cerebral dopamine neurotrophic factor precursor | gi|82880674 | MRVAELK | 16.69 | 864.29 | 6.58 | 1 | 6.58 | 0.002 |
| chemokine-like factor isoform c | gi|7705933 | LVFALVTAVCCLADGALIYR | 5.80 | 2239.62 | 4.52 | 1 | 4.52 | 0.008 |
| chromodomain-helicase-DNA-binding protein 2 isoform 1 | gi|118421089 | RHFSNADR | 8.89 | 996.78 | 4.69 | 1 | 4.69 | 0.007 |
| collagen alpha-1(XXIII) chain | gi|29725624 | GEKGAPGDFGPR | 8.43 | 1187.12 | 7.98 | 1 | 7.98 | 0.005 |
| cullin-9 | gi|24307991 | AAFMLALR | 5.60 | 918.58 | 4.68 | 1 | 4.68 | <0.001 |
| elongation factor 2 | gi|4503483 | VNFTVDQIR | 79.07 | 1100.35 | 4.91 | 1 | 4.91 | <0.001 |
| enolase family member 4 isoform 2 | gi|169201331 | RGQQQITGK | 13.54 | 1014.84 | 6.76 | 1 | 6.76 | 0.005 |
| fibroblast growth factor 10 precursor | gi|4758360 | SYNHLQGDVR | 6.01 | 939.69 | 5.36 | 1 | 5.36 | <0.001 |
| folliculin-interacting protein 1 | gi|72003827 | IGEPVWLTMMSGTPEK | 11.56 | 1791.12 | 8.75 | 1 | 8.75 | 0.005 |
| forkhead box P2 | gi|51095125 | QAIMESSDR | 5.67 | 1040.90 | 7.18 | 1 | 7.18 | 0.004 |
| guanine nucleotide-binding protein subunit alpha-12 | gi|42476111 | ILLLGAGESGK | 65.13 | 1057.81 | 7.84 | 1 | 7.84 | 0.005 |
| hCG1743503 | gi|119574355 | GAMAKPDCIITCDSK | 9.11 | 1466.86 | 6.04 | 1 | 6.04 | 0.006 |
| hCG1799966 | gi|119579543 | LSQDEHSFRGYR | 7.46 | 1493.57 | 7.84 | 1 | 7.84 | 0.009 |
| hCG1991899 | gi|119613961 | NIMNDWHLSLFISSR | 4.81 | 1848.99 | 8.38 | 1 | 8.37 | <0.001 |
| hCG2039006 | gi|119584274 | KTFNDTTLTFTHHIR | 2.38 | 1824.74 | 7.35 | 1 | 7.35 | 0.005 |
| hCG2045268 | gi|119590405 | TFAGLGSFSKEAQAR | 9.74 | 1569.09 | 6.86 | 1 | 6.86 | 0.010 |
| heterogeneous nuclear ribonucleoprotein A/B isoform a | gi|55956919 | IFVGGLNPEATEEK | 32.92 | 1504.11 | 6.91 | 1.33 | 5.21 | 0.004 |
| histone H2B | gi|1568557 | AMGIMNSFVNDIFER | 102.67 | 1776.00 | 7.78 | 1 | 7.78 | 0.009 |
| hydroxysteroid dehydrogenase-like protein 2 isoform 1 | gi|40254986 | DGANIVIAAKTAQPHPK | 3.04 | 1724.27 | 5.18 | 1 | 5.18 | 0.006 |
| hypothetical protein LOC286076 | gi|119602615 | DVGDALPR | 28.56 | 839.86 | 4.81 | 1 | 4.81 | 0.008 |
| immunoglobulin kappa chain | gi|5019513 | EYTFGQGTKLEIK | 15.40 | 984.11 | 5.66 | 1 | 5.66 | 0.005 |
| katanin p60 subunit A-like 1 | gi|55958971 | SEKPST | 6.67 | 658.45 | 5.05 | 1 | 5.05 | 0.002 |
| keratin 1b | gi|31074631 | FLEQQNQVLQTK | 65.33 | 1335.14 | 5.31 | 1 | 5.31 | 0.006 |
| keratin 25C | gi|119581082 | ALEEANADLEQKIK | 17.90 | 1565.14 | 5.47 | 1 | 5.47 | 0.003 |
| lamin-B1 isoform 1 | gi|5031877 | DAALATALGDKK | 58.67 | 1177.79 | 6.47 | 1 | 6.47 | 0.007 |
| microtubule-associated protein 6 isoform 2 | gi|48375167 | AGPAWIVR | 10.17 | 1078.13 | 5.94 | 1 | 5.94 | 0.006 |
| midasin | gi|24415404 | CRGFSAHLMK | 3.37 | 1207.69 | 5.30 | 1 | 5.30 | 0.009 |
| myosin B | gi|641958 | EQEVAELKK | 25.77 | 1074.84 | 4.92 | 1 | 4.92 | 0.006 |
| myotubularin-related protein 9 | gi|19923424 | MEFAELIK | 15.26 | 1003.57 | 7.02 | 1 | 7.02 | 0.003 |
| nucleolin | gi|55956788 | GFGFVDFNSEEDAK | 39.12 | 1562.07 | 6.74 | 1 | 6.74 | 0.008 |
| nucleoporin NYD-SP7 | gi|13508448 | DQEIEMR | 23.86 | 923.66 | 5.09 | 1 | 5.09 | 0.005 |
| pidermal growth factor-like protein 6 isoform 2 precursor | gi|268607667 | FGECVGPNK | 8.71 | 1006.74 | 7.17 | 1.54 | 4.67 | 0.009 |
| potassium voltage-gated channel subfamily KQT member 2 isoform a | gi|26051264 | GPLCGCCPGR | 9.11 | 1143.61 | 6.08 | 1 | 6.08 | 0.005 |
| regulator of G protein signaling 6 alpha1-GGL | gi|19908834 | KKPESEQGR | 5.93 | 1062.16 | 4.25 | 1 | 4.25 | 0.004 |
| regulator of G-protein signaling 5 isoform 1 | gi|4506519 | MAEKAK | 2.26 | 693.22 | 6.19 | 1 | 6.19 | 0.005 |
| tetratricopeptide repeat protein 24 | gi|282165719 | GLELLLR | 29.10 | 837.56 | 5.39 | 1 | 5.39 | 0.005 |
| tyrosine-protein kinase JAK2 | gi|4826776 | VKEPGESPIFWYAPESLTESK | 2.14 | 2393.40 | 7.91 | 1 | 7.91 | 0.004 |
| unnamed protein product | gi|34527379 | APPILSR | 23.71 | 759.49 | 5.34 | 1 | 5.34 | 0.007 |
| valosin-containing protein | gi|11095436 | KGDIFLVR | 18.33 | 950.23 | 6.56 | 1 | 6.56 | 0.005 |
| vinculin isoform VCL | gi|4507877 | AQQVSQGLDVLTAK | 53.99 | 1453.61 | 5.24 | 1 | 5.24 | <0.001 |
| zinc finger protein 423 | gi|46359075 | KGTQTSPVPR | 12.97 | 1071.70 | 7.67 | 1 | 7.67 | 0.002 |

* Rounded to two decimal places

** Rounded to three decimal places
